# Synthesis and Evaluation of Tricarbonyl ^99m^Tc-Labeled 2-(4-Chloro)phenyl-imidazo[1,2-*a*]pyridine Analogs as Novel SPECT Imaging Radiotracer for TSPO-Rich Cancer

**DOI:** 10.3390/ijms17071085

**Published:** 2016-07-07

**Authors:** Ji Young Choi, Rosa Maria Iacobazzi, Mara Perrone, Nicola Margiotta, Annalisa Cutrignelli, Jae Ho Jung, Do Dam Park, Byung Seok Moon, Nunzio Denora, Sang Eun Kim, Byung Chul Lee

**Affiliations:** 1 Department of Nuclear Medicine, Seoul National University College of Medicine, Seoul National University Bundang Hospital, Seongnam 13620, Korea; cjy0929@snu.ac.kr (J.Y.C.); jaehoboa@paran.com (J.H.J.); ddp2194@naver.com (D.D.P.); bsmoon@snu.ac.kr (B.S.M.); kse@snu.ac.kr (S.E.K.); 2 Department of Transdisciplinary Studies, Graduate School of Convergence Science and Technology, Seoul National University, Seoul 16229, Korea; 3 Department of Pharmacy–Drug Sciences, University of Bari “Aldo Moro”, Bari 70125, Italy; rosamaria.iacobazzi@uniba.it (R.M.I.); mara.perrone@uniba.it (M.P.); annalisa.cutrignelli@uniba.it (A.C.); 4 Istituto Tumori IRCCS “Giovanni Paolo II”, Flacco, St. 65, Bari 70124, Italy; 5 Department of Chemistry, University of Bari “Aldo Moro”, Bari 70125, Italy; nicola.margiotta@uniba.it; 6 Center for Nanomolecular Imaging and Innovative Drug Development, Advanced Institutes of Convergence Technology, Suwon 16229, Korea

**Keywords:** translocator protein, TSPO, Tricarbonyltechnetium-99m, ^99m^Tc(CO)_3_, TSPO-rich tumors, SPECT

## Abstract

The 18-kDa translocator protein (TSPO) levels are associated with brain, breast, and prostate cancer progression and have emerged as viable targets for cancer therapy and imaging. In order to develop highly selective and active ligands with a high affinity for TSPO, imidazopyridine-based TSPO ligand (CB256, **3**) was prepared as the precursor. ^99m^Tc- and Re-CB256 (**1** and **2**, respectively) were synthesized in high radiochemical yield (74.5% ± 6.4%, decay-corrected, *n* = 5) and chemical yield (65.6%) by the incorporation of the [^99m^Tc(CO)_3_(H_2_O)_3_]^+^ and (NEt_4_)_2_[Re(CO)_3_Br_3_] followed by HPLC separation. Radio-ligand **1** was shown to be stable (>99%) when incubated in human serum for 4 h at 37 °C with a relatively low lipophilicity (log*D* = 2.15 ± 0.02). The rhenium-185 and -187 complex **2** exhibited a moderate affinity (*K*_i_ = 159.3 ± 8.7 nM) for TSPO, whereas its cytotoxicity evaluated on TSPO-rich tumor cell lines was lower than that observed for the precursor. In vitro uptake studies of **1** in C6 and U87-MG cells for 60 min was found to be 9.84% ± 0.17% and 7.87% ± 0.23% ID, respectively. Our results indicated that ^99m^Tc-CB256 can be considered as a potential new TSPO-rich cancer SPECT imaging agent and provides the foundation for further in vivo evaluation.

## 1. Introduction

The 18-kDa translocator protein (TSPO) is a mitochondrial five transmembrane protein, which is principally located in the outer mitochondrial membrane and associated with a wide number of biological processes including cell proliferation, apoptosis, steroidogenesis, and immunomodulation [1,2,3,4]. Moreover, elevated TSPO levels are well documented in oncology and have been correlated with tumor proliferation, invasion, and metastasis including brain, colorectal, liver, breast, oral cavity, and prostate carcinomas [5,6,7]. Thus, TSPO is a suitable imaging target for both inflammatory neurodegenerative diseases and cancer because it is highly expressed in activated microglial cells and surrounding TSPO-rich tumors, but absent in normal healthy tissues, except for kidney, heart, and gonads. Over the years, TSPO-specific ligands have been widely investigated and shown to be valuable tools for targeting the progression of pathologies associated with overexpression of TSPO. Various TSPO ligands are known from many different structural classes such as isoquinoline carboxamides (e.g., PK 11195), benzodiazepines (e.g., Ro-54864), phenoxyarylacetamides (e.g., DAA1106), aryloxyanilides (e.g., PBR28) and 2-phenyl-imidazo[1,2-*a*]pyridine acetamides (e.g., alpidem) [8,9,10,11,12]. Although a wide number of TSPO ligands have been developed for positron emission tomography (PET) and single photon emission computed tomography (SPECT) imaging, few ^99m^Tc-labeled ligands have been reported so far [13,14,15,16]. Among the radionuclides, ^99m^Tc is desirable due to its ideal physical properties (γ-emission of 141 keV, *t*_1/2_ = 6 h) for imaging purposes [17,18]. Moreover, ^99m^Tc is readily obtained by daily elution from ^99^Mo/^99m^Tc-generator and thus, it is very convenient and suitable for routine clinical use. In addition, the tricarbonyl technetium-99m (^99m^Tc-(CO)_3_) unit has been shown to be useful for introducing ^99m^Tc into biomolecules because of its high chemical stability and small size. In order to take advantage of molecular imaging techniques with ^99m^Tc-(CO)_3_, many low-molecular weight ^99m^Tc(I)-complex with tridentate ligands have been developed and used for the preparation of ^99m^Tc-(CO)_3_-labeled radiotracers [19,20,21,22,23,24].

In our previous studies, potent and selective imidazopyridine-based TSPO ligands—which could carry both a cytostatic platinum species and a rhenium complex as the precursor for introducing the ^99m^Tc-(CO)_3_ unit—were reported [25,26,27,28,29,30]. Among the imidazopyridine-based TSPO ligands investigated so far, 2-(8-(2-(bis(pyridin-2-yl)methyl)amino)acetamido)-2-(4-chlorophenyl)*H* imidazo [1,2-*a*] pyridin-3-yl)-*N*,*N*-dipropylacetamide (CB256, **3**) contains the TSPO-targeting moiety and a metal ion anchor, namely di-(2-picolyl)amine, for a bifunctional chelate approach [29].

Our goal was to exploit the imidazopyridine-based ligand CB256 for incorporating the tricarbonyl ^99m^Tc radioisotope to obtain a new TSPO-selective imaging agent. In addition, Re-CB256 was prepared as a model of ^186/188^Re-CB256, a potential TSPO-targeted internal radiation therapy agent.

## 2. Results and Discussion

### 2.1. Synthesis of Re-CB256 and ^99m^Tc-CB256

The precursor **3** (CB256) was prepared from the TSPO ligand CB86 and bromoacetyl bromide in the presence of triethylamine, followed by *N*-alkylation with di-(2-picolyl)amide, according to a previously described method [29]. The coordination potential of CB256 towards Pt(II) and Re(I) metal ions was already exploited by some of us and, in particular, two dinuclear Pt/Re and Re/Re complexes were prepared, indicating that the introduction of the di-(2-picolyl)amine moiety allows the coordination of a metal ion such as Pt^2+^ or Re^+^ [30]. Unlike our previous results of the dinuclear Re complex, only the homologous ^99m^Tc complex was generated in the radiolabeling reaction due to the low concentration of technetium. The cold Re-CB256 (**2**) was prepared to identify the chemical characteristics of ^99m^Tc-CB256 based on the similar chemical properties between Tc and Re complex. The coordination of Re to the imidazopyridine residue reduced the affinity of CB256 towards TSPO, hence, in this investigation, we have sought to coordinate a single metal ion to the di-(2-picolyl)amine chelate residue to obtain a diagnostic drug (^99m^Tc in compound **1**) or a model of a therapeutic drug (using cold Re in compound **2**).

**Scheme 1:**
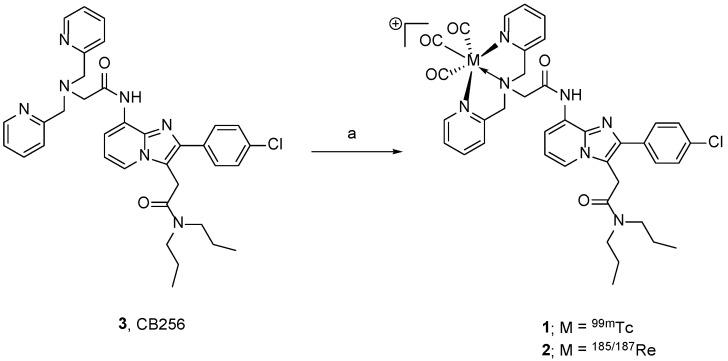
Synthesis of ^99m^Tc- and Re-CB256. Reagents and conditions: (a) [^99m^Tc(CO)_3_(H_2_O)_3_]^+^, MeOH–H_2_O, 65 °C, 0.5 h or (NEt_4_)_2_[Re(CO)_3_Br_3_], MeOH, 65 °C, 1 h.

**Figure 1:**
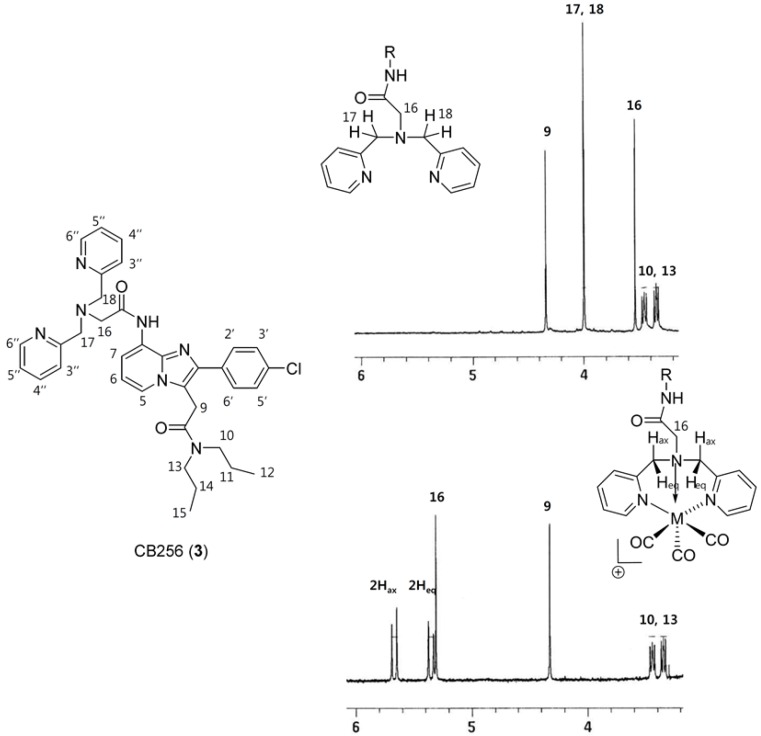
Section of the ^1^H-NMR spectra between 6.0 and 3.2 ppm of the precursor **3** (**upper**) and of the “cold” rhenium complex **2** (**bottom**).

As shown in Scheme 1, the “cold” rhenium complex **2** was prepared by treating the TSPO ligand **3** in methanol at 65 °C with (NEt_4_)_2_[ReBr_3_(CO)_3_]. The reaction was monitored by HPLC until the precursor peak (*t*_R_ = 14.5 min) disappeared. The desired Re(CO)_3_ core, coordinated to CB256 (**2**), was obtained in good yield (60%–71%) and revealed a HPLC retention time of 22 min. Compound **2** was characterized by ^1^H-NMR (Figure 1) and ^13^C-NMR spectroscopy and by HRMS (ESI).

The coordination of the ^99m^Tc radioisotope to the di-(2-picolyl)amine moiety of the TSPO-ligand **3** was obtained in aqueous media by using the *fac*-[^99m^Tc(H_2_O)_3_(CO)_3_]^+^ synthon, which can be readily generated from ^99m^TcO_4_^−^ and CO gas in the presence of NaBH_4_ [20,31].

**Figure 2:**
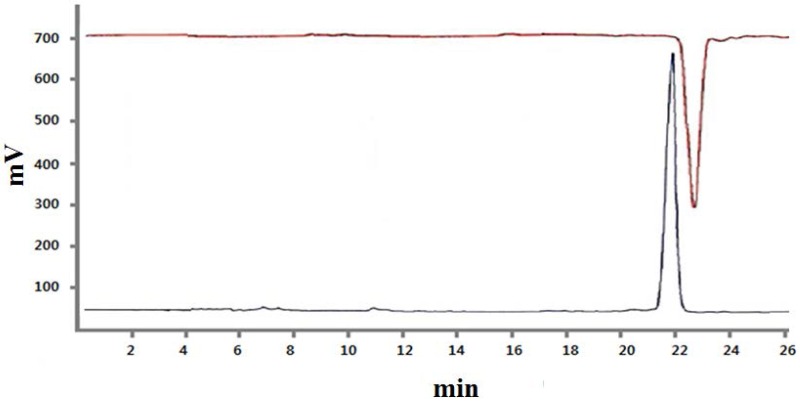
HPLC chromatograms of ^99m^Tc-CB256 (**1**, red) and Re-CB256 (**2**, black). Xterra RP-18; 20%–90% acetonitrile-water; flow rate 3 mL/min.

The radiochemical yield and purity of **1** were 74.5% ± 6.4% (decay-corrected) and >95%, respectively. Compound **1** was characterized by HPLC (Figure 2) by comparison with the chromatogram of **2**. The retention times for **1** and **2** were found to be 22.5 and 22 min, respectively.

### 2.2. In Vitro Stability Studies and Partition Coefficient of ^99m^Tc-CB256 (**1**)

The percentage of **1** remaining in solution after 4 h of incubation in human serum at 37 °C was 99% as calculated by radio-TLC scanner, indicating a high in vitro stability of the radiotracer. The partition coefficient of **1** (Log*D* = 2.15 ± 0.02 vs. 1.08 ± 0.02 for **3**) indicated a relatively low lipophilicity compared to that of fluorine-substituted imidazo [1,2-*a*]pyridine acetamide analogs (i.e., 3.00 ± 0.03 for [^18^F]CB251) prepared in our previous investigations [32]. However, the lower lipophilicity did not prevent the uptake of **1** in tumor cells (see Section 2.3).

### 2.3. In Vitro Cell Uptake Assay of ^99m^Tc-CB256 (**1**)

**Figure 3:**
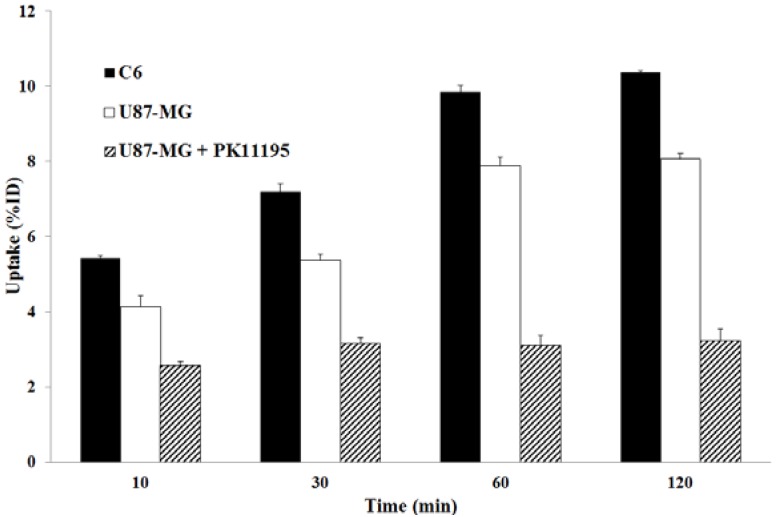
Uptake kinetic of ^99m^Tc-CB256 (**1**) into C6 and U87-MG cells in the presence or absence of PK 11195. Data are expressed as percentage injected dose (% ID, mean with S.D. *n* = 4). Closed bar: uptake of **1** in C6 cells; open bar: uptake of **1** in U87-MG cells; right-handed striped bar: uptake of **1** in U87-MG cells in the presence of 300 µM PK 11195.

The in vitro uptake of **1** by tumor cells was measured in two different cancer cell lines overexpressing the TSPO receptor, namely C6 rat glioma and U87-MG human glioblastioma cell lines. The results are shown in Figure 3 and indicate that the uptake of **1** was time-dependent and reached almost the highest level after 60 min of incubation (9.84% ± 0.17% and 7.87% ± 0.23% ID in C6 and U87-MG cells, respectively). This result is a direct consequence of the lipophilicity of compound **1**. In blocking experiments conducted on U87-MG cells in the presence of the TSPO ligand PK 11195, the cell uptake of **1** was markedly decreased throughout the experimental period and the observed relative uptake reduction was 63.5%. These displacement studies indicate that the uptake of **1** in the tumor cells was selectively and specifically mediated by TSPO, and support the potential use of compound **1** as TSPO marker for SPECT diagnosis.

### 2.4. In Vitro Cell Binding Affinity of CB256 (**3**) and Re-CB256 (**2**)

The affinity for TSPO of compounds **3** and **2** was evaluated by measuring their ability to displace the reference compound [^3^H]-PK 11195 from the membrane extracts of C6 glioma cells. The results show that the free TSPO ligand **3** has an appreciable affinity for TSPO (148 nM), which, however, is lower than that of the reference compound PK 11195 (9 nM). This result is in agreement with that previously reported [30], and the reduced affinity of compound **3** could be explained by the steric bulk generated by the dipicolylaminic moiety at position 8 of the imidazopyridine nucleus, which is crucial for the interaction of the ligand with mitochondrial TSPO [29,30]. As expected, compound **2** showed an affinity (159 nM) comparable to that of the free ligand (Table 1). In fact, as already reported, the coordination of a metal ion (Pt(II)) to the tridentate bis-(2-picolyl)amine residue did not significantly alter the TSPO affinity of CB256, while metalation at the imidazopyridine moiety greatly reduced the affinity for TSPO [30]. Even though the affinity of compounds **2** and **3** are lower to that of the reference compound PK 11195, these binding values can be still good for biological applications.

### 2.5. In Vitro Cytotoxicity Assays of CB256 (**3**) and Re-CB256 (**2**)

Table 2 summarizes the cytotoxicity of **2** and **3** against HepG2, MCF7, and U87 cancer cells exposed for a period of 72 h. Our previous investigation has shown that compound **3** is extremely effective toward C6 glioma cells [29]. The high cytotoxicity of **3** was correlated with its ability to produce double-strand lesions on DNA after coordination of a biometal, such as Cu(I) [29]. In the present study, compound **3** was found to be cytotoxic against HepG2, MCF7, and U87 cancer cells, confirming the above-mentioned evidence. On the contrary, as expected, compound **2** has much lower cytotoxicity. The lower cytotoxicity of Re-CB256 with respect to uncoordinated CB256 can be explained by its inability to coordinate a biometal (CuI) and therefore to act as a double-strand breaker of DNA.

## 3. Experimental Section

### 3.1. Materials and Methods

All commercial reagents and solvents were used without further purification unless otherwise specified. Reagents and solvents were purchased from Sigma-Aldrich and TCI. ^1^H- and ^13^C-NMR spectra were recorded on a Varian at 400-MR (400 MHz) spectrometer (Agilent Technologies, Santa Clara, CA, USA) at ambient temperature. Chemical shifts were reported in parts per million (ppm, δ units). Electrospray mass spectrometry (ESI-MS) was performed on a LC/MS spectrometer (Agilent 6130 Series, Agilent Technologies). HPLC was carried out on a Thermo Separation Products System (Fremont, CA, USA) equipped with a semi-preparative column (Waters, Xterra RP-C18, 10 µm, 10 × 250 mm) and equipped with a UV detector (wavelength set at 254 nm) and a γ-ray detector (Bioscan, Poway, CA, USA). HPLC-grade solvents (J. T. Baker, Phillipsburg, NJ, USA) were used for HPLC purification after membrane filtering (Whatman, Maidstone, UK, 0.22 µm). The column was eluted with a solvent mixture of acetonitrile-water (0.1% trifluoroacetic acid) using a gradient condition. The HPLC eluent started with 20% acetonitrile-water (0.1% trifluoroacetic acid) and the ratio was increased with a solvent mixture of 90% acetonitrile-water (0.1% trifluoroacetic acid) over 30 min at a flow rate of 3 mL/min. TLC was performed on Merck F254 silica plates and radio-TLC was analyzed on a Bioscan radio-TLC scanner (Washington, DC, USA). All radioactivities were measured using a VDC-505 activity calibrator from Veenstra Instruments (Joure, The Netherlands). In vitro incubation was carried out at 37 °C using a block heater (Digi-Block Laboratory Device Inc., Holliston, MA, USA). Na^99m^TcO_4_ was eluted on a daily basis from ^99^Mo/^99m^Tc generators (Samyoung Unitech, Seoul, Korea). The organometallic precursor (NEt_4_)_2_[ReBr_3_(CO)_3_] and the radioactive precursor [^99m^Tc(CO)_3_(H_2_O)_3_]^+^ were prepared as previously reported [29,31,33].

### 3.2. Synthesis of Re-CB256 (**2**)

A solution of **3** (2 mg, 3 µmol) in methanol (1 mL) was treated with (NEt_4_)_2_[ReBr_3_(CO)_3_] (2.3 mg, 3 µmol). The reaction mixture was stirred at 65 °C for 1 h. The solvent was removed under reduced pressure and then the product was separated by a semi-preparative HPLC system. The fraction of **2** was collected at 22 min as yellow solid: m.p. 190.3–210.4 °C; ^1^H-NMR (400 MHz, acetone-D_6_) δ 9.02 (d, *J* = 5.2 Hz, 2H, H_6′′_), 8.27 (d, *J* = 6.8 Hz, 1H, H_7_), 8.10 (t, *J* = 7.6 Hz, 2H, H_3′′_), 8.07 (d, *J* = 6.0 Hz, 1H, H_5_), 7.81 (d, *J* = 8.0 Hz, 2H, H_2′_/H_6′_), 7.72–7.69 (m, 2H, H_4′′_), 7.55 (d, *J* = 6.8 Hz, 2H, H_3′_/H_5′_), 7.53 (m, 2H, H_5′′_), 6.99 (t, *J* = 7.2 Hz, 1H, H_6_), 5.69 (d, *J* = 17.2 Hz, 2H, H_17(ax)/_H_18(ax)_), 5.37 (d, *J* = 17.2 Hz, 2H, H_17(eq)/_H_18(eq)_), 5.31 (s, 2H, H_16_), 4.32 (s, 2H, H_9_), 3.44 (t, *J* = 7.6 Hz, 2H, H_10_), 3.34 (t, *J* = 7.6 Hz, 2H, H_13_), 1.73–1.67 (m, 4H, H_11_/H_14_), 0.92–0.84 (m, 6H, H_12_/H_15_); ^13^C-NMR (100 MHz, acetone-D_6_) δ 197.1 (overlap with CO and CONH), 196.7, 169.4, 168.7, 163.0, 153.6, 142.2, 135.1, 131.5, 130.3, 127.5, 125.4, 122.2, 120.0, 114.2, 106.6, 71.7, 70.0, 51.0, 49.0, 23.6, 22.3, 12.3, 12.1; MS (ESI) *m*/*z* 894.2 (M^+^, 100%), 892.2 (53%), 895.2 (40%). HRMS (ESI) *m*/*z* C_38_H_38_O_5_N_7_ClRe calcd: 894.2175; found: 894.2155.

### 3.3. Synthesis of ^99m^Tc-CB256 (**1**)

A solution of [^99m^Tc(H_2_O)_3_(CO)_3_]^+^ in saline (250 µL, approximately 44 MBq) was added to a solution of **3** (1 mg, 1.5 µmol) dissolved in methanol (250 µL). The reaction mixture was stirred at 65 °C for 20 min. After the reaction time, the mixture was cooled in an ice-bath and diluted with 10 mL of water. This solution was loaded into a C18 Sep-Pak cartridge, washed with 5 mL of water, and eluted with 1.5 mL of acetonitrile. The combined solvent fractions were removed by a stream of nitrogen gas. The product was purified by a semi-preparative HPLC system. The radiochemically pure **1** eluted off with a retention time of 22.5 min, and the radiochemical yield, calculated from a homemade [^99m^Tc(H_2_O)_3_(CO)_3_]^+^ solution in saline, was 74.5% ± 6.4% (decay-corrected). The obtained **1** was diluted with excess water, passed through a C18 Sep-Pak cartridge and washed with water (5 mL). The desired product was eluted by ethanol (1.5 mL) and exchanged to 10% ethanol-saline for in vitro experiments. The identity was confirmed by coinjection with authentic compound **2** as shown in Figure 2.

### 3.4. In Vitro Stability Study

The stability of **1** was assayed by monitoring the Radio-TLC profile and determining its radiochemical purity. Human serum was prepared from human whole blood by centrifuging at 3500 rpm for 5 min. An aliquot (3.7 MBq) of **1** in 10% ethanol-saline (0.1 mL) was added to human serum (0.5 mL) and incubated at 37 °C for 4 h. At the indicated time points (10, 30, 60, 120, and 240 min), the sample was taken and then added to acetonitrile (0.1 mL). After vortexing (20 s), the mixture was centrifuged at 3500 rpm for 5 min. The obtained supernatant was analyzed by radio-TLC using methanol-dichloromethane (1:9, *R*_f_ = 0.6 for **1**) as the developing solvents.

### 3.5. LogD Determination

The Log*D* value was measured by mixing a solution of **1** in 5% ethanol-saline (10 µL, approximately 0.74 MBq) with sodium phosphate buffer (0.15 M, pH 7.4, 5 mL) and *n*-octanol (5 mL) in a test tube. After vortexing for 1 min, each tube was then stored for 3 min at room temperature and the phases were separated. Samples of each phase (100 µL) were counted for radioactivity. Log*D* is expressed as the logarithm of the ratio of the counts from *n*-octanol versus that of the sodium phosphate buffer.

### 3.6. In Vitro Cell Uptake Assay of ^99m^Tc-CB256 (**1**)

Rat C6-glioma cells (C6) and human glioblastoma U87-MG cells were purchased from the American Type Culture Collection (ATCC). C6 cells were cultured in Dulbecco’s Modified Eagle’s Medium (DMEM) containing high glucose (WelGENE), supplemented with 10% heat-inactivated fetal bovine serum (FBS; GIBCO) and antibiotics (100 units/mL penicillin G and 10 µg/mL reptomycin; GIBCO) at 37 °C in a humidified 5% CO_2_ atmosphere. U87-MG cells were cultured in DMEM supplemented with 4 mM l-glutamine, 4500 mg/L glucose, 1 mM sodium pyruvate, 1.5 g/mL sodium bicarbonate, 10% FBS, and antibiotics-antimycotic at 37 °C in a humidified 5% CO_2_ atmosphere. Both cells (1 × 10^6^ cells in 0.1 mL medium per test tubes) were incubated with ^99m^Tc-CB256 (**1**) (0.74 MBq in 0.1 mL 10% ethanol-saline) at 10, 30, 60, and 120 min. In an inhibition study for specificity, ^99m^Tc-CB256 involved with 300 µM PK 11195 was performed in U87-MG cells. After incubation, the cells were quickly washed twice (<15 s) with 1 mL of ice-cold phosphate buffer saline and centrifuged at 3500 rpm for 5 min. Each supernatant was removed for counting, and the remaining sample containing cells was measured by a γ counter (1480 WIZARD, Perkin-Elmer). ^99m^Tc uptake was expressed as the percentage injected dose (% ID).

### 3.7. In Vitro Cell-Binding Assays

Binding affinity and selectivity to the 18-kDa translocator protein TSPO and to CBR were assessed using in vitro receptor-binding assays. These experiments were carried out as previously described [28].

### 3.8. Cytotoxicity Assays

Cytotoxicity assays were carried out against HepG2, MCF7, and U87 cancer cells seeded at a density of 5000 cells/well. All tested compounds were dissolved in DMSO prior to their dilution with cell culture medium to the predetermined experimental concentrations (eight concentrations ranging from 0.01 to 50 µM), with the final DMSO concentration never exceeding 1%. Cytotoxicity (IC_50_) values for the tested compounds were determined using the 3-(4,5-dimethylthiazol-2-yl)-2,5-diphenyltetrazolium bromide (MTT) assay. Briefly, the cells were seeded in a 96-well plate and incubated at 37 °C for 72 h with the tested compounds. Then, 10 µL of 5 mg/mL MTT were added to each well and the plates were incubated for an additional 4 h at 37 °C. Subsequently, cells were lysed by addition of 150 µL of 50% (*v*/*v*) DMSO and 50% (*v*/*v*) ethanol solution, and the absorbance of each individual well was measured using a microplate reader at 570 nm (Wallac Victor3, 1420 Multilabel Counter, Perkin-Elmer (manufactured for WALLAC Oy, Turku, Finland)). The reported values are the average of triplicate measurements performed in at least three separate experiments.

## 4. Conclusions

A ^99m^Tc-labeled imidazopyridine-based bifunctional chelate ligand (**1**) was prepared in one step by coordination of the tricarbonyl ^99m^Tc core to the di-(2-picolyl)amine residue, with good radiochemical yield. The resulting complex (**1**) showed high stability in vitro. The affinity toward TSPO of **2** proved that the tricarbonyl rhenium moiety did not alter the TSPO affinity of CB256 (**3**). The low cytotoxicity of **1** further demonstrates that if the dipicolylamine moiety is coordinated to a metal ion—in the present case the tricarbonyl Re-core—it is not able to bind endogenous biometals to exert its DNA cleavage activity and cause double-strand DNA lesions. In vitro studies on TSPO-rich tumor cells suggest that radiolabeled **1** may have potential to act as a useful SPECT radiotracer for the evaluation of TSPO-overexpressing tissues, and provides the foundation for further in vivo biological evaluation.

## Figures and Tables

**Table 1:** Affinities (*K*_i_/nM) of Re-CB256 (**2**) for TSPO from rat C6 glioma cells membranes. Corresponding values for PK 11195 and CB256 (**3**) are also reported for comparison.

Compound	*K*_i_ (nM) for TSPO
PK 11195	9.10 ± 1.2
CB256 (**3**)	148.2 ± 11.3
Re-CB256 (**2**)	159.3 ± 8.70

**Table 2:** Cytotoxicity of CB256 (**3**) and Re-CB256 (**2**) toward HepG2, MCF7 and U87 cancer cell lines.

Compound	IC_50_ (µM) ^a^		
	HepG2	MCF7	U87
CB256 (**3**)	30 ± 5	38 ± 3	35 ± 2
Re-CB256 (**2**)	>50 (67%) ^b^	>50 (56%) ^b^	>50 (59%) ^b^

^a^ Cells were seeded at a density of ~5000 cells per well into 96-well plates. Following overnight incubation, cells were treated with a range of drug concentrations (from 0.01 to 50 µM) and incubated at 37 °C under a humidified atmosphere with 5% CO_2_ for a period of 72 h. Data are the mean values ± SD of three independent experiments performed in triplicate; ^b^ In parenthesis the percentage of cell viability at highest tested concentration (50 µM).

## Abbreviations

TSPO	18-kDa mitochondrial translocator protein
PET	positron emission computed tomography
SPECT	single photon emission computed tomography
HPLC	high performance liquid chromatography
ESI-MS	electrospray mass spectrometry
HRMS	high resolution mass spectrometry
CBR	central benzodiazephine receptor
U87-MG	U87-malignant glioma
% ID	% injected dose
NMR	nuclear magnetic resonance
TLC	thin layer chromatography
S.D.	standard deviation
